# Putting on molecular weight: Enabling cryo-EM structure determination of sub-100-kDa proteins

**DOI:** 10.1016/j.crstbi.2022.09.005

**Published:** 2022-10-02

**Authors:** Koen Wentinck, Christos Gogou, Dimphna H. Meijer

**Affiliations:** Department of Bionanoscience, Kavli Institute of Nanoscience Delft, Delft University of Technology, van der Maasweg 9, 2629HZ, Delft, the Netherlands

**Keywords:** BRIL, cytochrome*b*562 RIL, cryo-EM, cryogenic electron microscopy, DARPin, Design Ankyrin Repeat Protein, Fab, antigen binding fragment, GFP, Green Fluorecent Protein, GPCR, G protein-coupled receptor, kDa, kiloDalton, Mb, megabody, MW, molecular weight, Nb, nanobody, SPA, single particle analysis, SNR, signal-to-noise ratio, TM, transmembrane, κOR ICL3, κ-opiod receptor intracellular loop 3

## Abstract

Significant advances in the past decade have enabled high-resolution structure determination of a vast variety of proteins by cryogenic electron microscopy single particle analysis. Despite improved sample preparation, next-generation imaging hardware, and advanced single particle analysis algorithms, small proteins remain elusive for reconstruction due to low signal-to-noise and lack of distinctive structural features. Multiple efforts have therefore been directed at the development of size-increase techniques for small proteins. Here we review the latest methods for increasing effective molecular weight of proteins <100 ​kDa through target protein binding or target protein fusion - specifically by using nanobody-based assemblies, fusion tags, and symmetric scaffolds. Finally, we summarize these state-of-the-art techniques into a decision-tree to facilitate the design of tailored future approaches, and thus for further exploration of ever-smaller proteins that make up the largest part of the human genome.

## 1 Introduction

**Fig. 1:**
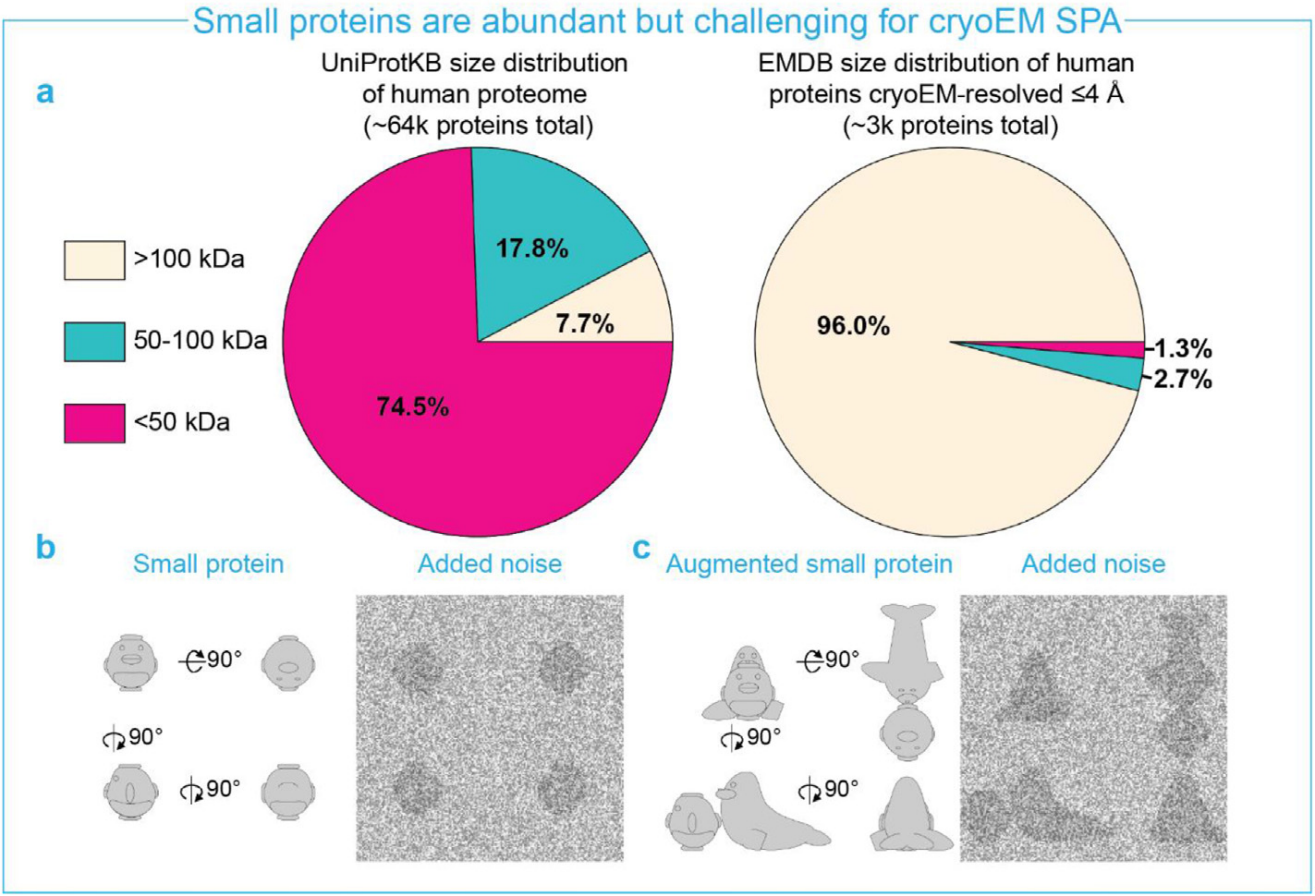
**a.** Size distribution of the UniProtKB human proteome compared to size distribution of EMDB entries of human proteins resolved below 4 ​Å resolution. Small proteins (<100 ​kDa) are under-represented in the SPA electron density database relative to their known abundance in the human proteome **b.** Low signal-to-noise ratio and lack of morphological features of small proteins (depicted as penguins) in electron micrographs complicates proper alignment of particles. **c.** Augmenting small proteins by appending extra molecular weight (depicted as seals) increases the signal-to-noise ratio and adds distinctive features to the assembly. The appended asymmetric body (missing flipper of the seal) allows different orientations of the assembly to be distinguishable for unambiguous alignment.

In the last decade, cryogenic electron microscopy (cryo-EM) single particle analysis (SPA) has become a prominent method in structural biology for protein structure determination (Renaud et al., 2018; Wigge et al., 2020). Compared to other complementary structural methods such as X-ray crystallography and NMR, cryoEM SPA has significant advantages: It is compatible with large, heterogenous macromolecular protein complexes in intact membrane environments, and it also typically requires much lower sample concentrations (Overington et al., 2006; García-Nafría and Tate, 2020). However, high-resolution structure determination of small proteins (<100 ​kDa) remains difficult using cryo-EM SPA. To illustrate, the current structures of human proteins on the electron microscopy data bank (EMDB) below 100 ​kDa and better than 4.0 ​Å resolution covers less than 3% of the total number of human-protein entries. In contrast, nearly 75% of the known human proteome members are smaller than 50 ​kDa (Brocchieri and Karlin, 2005) (Fig. 1a). High-resolution reconstruction of small proteins from cryo-electron micrographs is problematic for two main reasons (Fig. 1b-c): (i) Small proteins typically suffer from a lower signal-to-noise ratio (SNR), as the SNR depends on the vitreous ice thickness relative to the particle size (Yonekura et al., 2006; Himes and Grigorieff, 2021). This is particularly hard to optimize for smaller molecules. (ii) Small proteins intrinsically have few distinctive morphological features, which complicates particle alignment needed for protein reconstructions (Lander and Glaeser, 2021). The inability to resolve small protein structures is currently a serious limitation for the general applicability of cryo-EM.

Despite great improvements in particle reconstruction software and electron microscopy hardware – such as direct detectors, energy filters, and phase plates – low SNR and lack of distinctive morphological features remain an impairment for faithful reconstruction of small protein targets (Nygaard et al., 2020). An elegant solution for enabling structure determination of small proteins with cryo-EM would then be to increase the molecular weight (MW) of the target protein. Here we review recent endeavors to overcome these size-dependent limitations by effectively increasing target protein size through tightly attached proteinaceous augmentations. Tight attachment is essential, as flexibly attached augmentations typically result in reduced resolution of the reconstructed molecule. We focus on techniques that are adaptable to a wide range of small proteins and categorize them in ‘target protein binding’ and ‘target protein fusion’ methods to distinguish between their mode of implementation. The resulting oligomers or chimeras, respectively, directly increase target protein size to improve SNR, while the added MW also serves as a fiducial marker for particle localization and alignment. Ideally, such fiducials add asymmetric features to reduce ambiguity in the alignment of differently oriented particles.

Additionally, examples from both categories will be addressed in which augmentations form higher-order symmetrical scaffolds, further facilitating high-resolution reconstructions of ever smaller proteins. Finally, we offer an overview of requirements, considerations, and suggestions to assist the design of future small-protein studies toward generalized applicability of size-increase techniques for SPA.

## 2 Target binding strategies

To append MW to a target protein without direct modification of the target protein itself, several approaches have been developed. Non-fusion size-increase for cryo-EM SPA has been realized using scaffolding DNA (Aissaoui et al., 2021), thermostabilized antagonists (Zhang, 2022b), ubiquitin (Chiu et al., 2021), antigen-binding fragments (Fabs) (Wu et al., 2012; Coleman et al., 2020; Nygaard et al., 2020), nanobodies (Uchański et al., 2020), and Design Ankyrin Repeat Proteins (DARPins) (Liu et al., 2018; Yao et al., 2019; Yeates et al., 2020; Vulovic et al., 2021). The three latter options are most promising for general applicability since they are compatible with *in vitro* display technologies (Helma et al., 2015) to obtain high affinity binders against a wide range of target proteins and will be discussed in more detail below.

As early as 2012, Fabs were proposed to serve as fiducial markers for alignment (Wu et al., 2012). Fabs are ∼50 ​kDa proteins and have a very characteristic “elbow” shape. In cryo-EM, Fabs have been proven to be very successful, especially for resolving small membrane proteins that often lack a characteristic feature when embedded in a detergent micelle or nanodisc. This topic has been excellently covered by Nygaard and colleagues (Nygaard et al., 2020). Unfortunately, a stable and rigid Fab is not commercially available for every protein, and a single Fab might still add insufficient MW and features for structure determination.

**Fig. 2:**
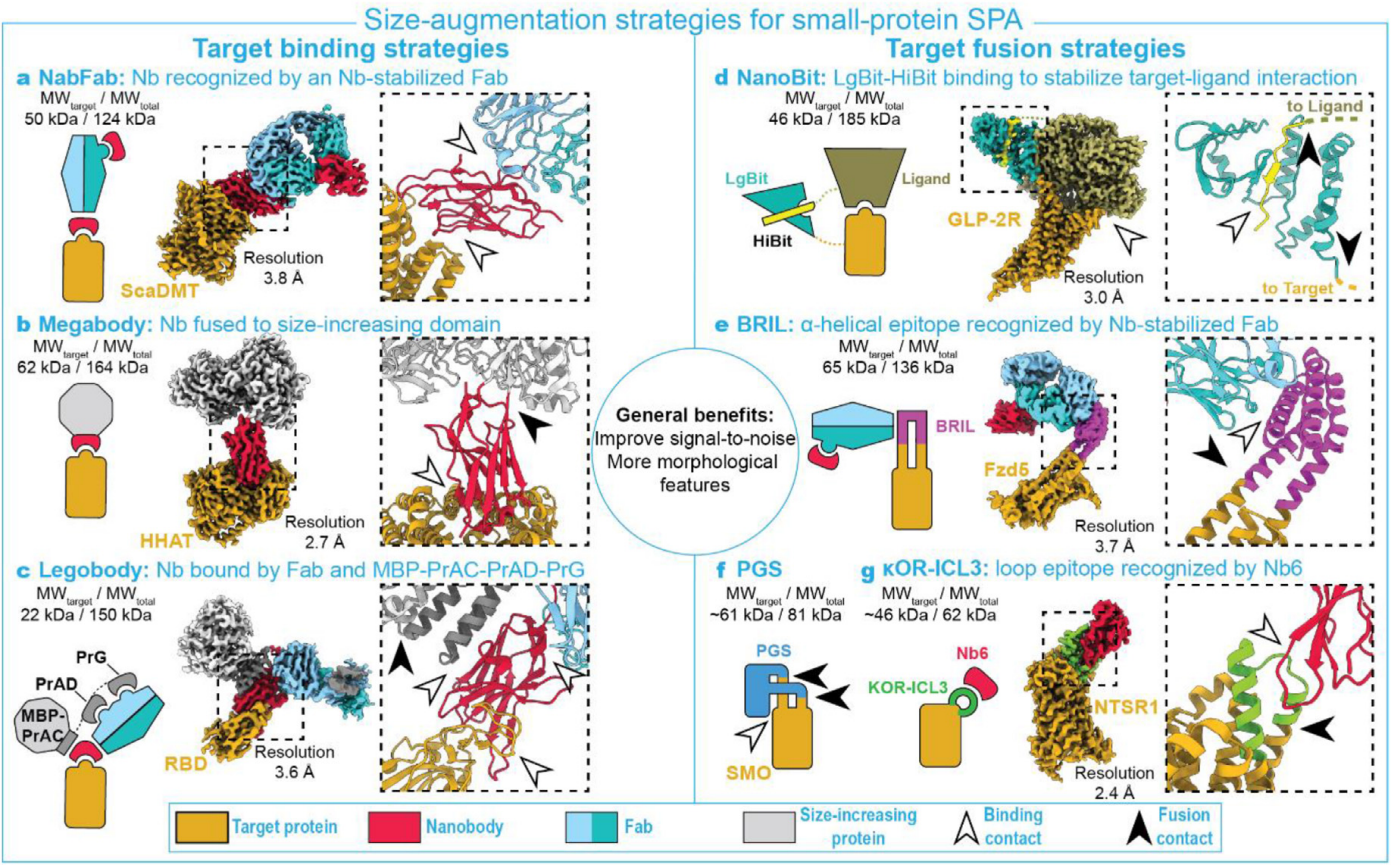
**a.** NabFab enabled 3.8 ​Å-resolution reconstruction of 50-kDa target protein ScaDMT (EMD-13438, PDB:7PIJ). The target is rigidly bound by a Nb, which in turn is bound by a Nb-specific Fab. An additional Nb is bound to minimize the Fab internal flexibility. **b.** Example of a Megabody enabling a 2.7 ​Å-resolution reconstruction of 62-kDa target protein HHAT (EMD- 13764, PDB:7Q1U). A Mb, consisting of a target-specific Nb fused with a large protein, is stably bound to the target protein. **c.** Use of a Legobody enabled 3.6 ​Å-resolution reconstruction of 22-kDa target protein SARS-CoV-2 RBD (EMD-24729, PDB:7RXD). The target is rigidly bound by a Nb, which in turn is bound by a Nb-specific Fab. This assembly is stabilized by a MBP-PrAC fused to Fab-binding PrAD-PrG which binds Nb as well as Fab. **d.** Nanobit enabled high-resolution reconstruction of 46-kDa target protein GLP-2R (EMD-30590, PDB:7D68). LgBit-fused GPCR is bound to its HiBit-fused natural G protein ligand. The LgBit-HiBit interaction stabilizes the physiological interaction between GPCR and G protein. The originally unresolved LgBit and HiBit structures were predicted using AlphaFold (Jumper et al., 2021). NanoBit schematic not to scale. **e.** A so-called BRIL insert enabled 3.7 ​Å-resolution reconstruction of 65-kDa target protein Fzd5 (EMD-21927, PDB:6WW2). BRIL is a two-point insertion between two anti-parallel α-helices of the target protein. BRIL serves as binding site for a Nb-stabilized Fab. **f.** Schematic of a PGS-insertion strategy that enabled the 3.7 ​Å-resolution reconstruction of ∼61-kDa target protein SMO. PGS is fused to the target protein via a flexible two-point α-helical insertion, whereby the structural rigidity of the target-PGS fusion is ensured by a hydrophobic interaction between the target and PGS. **g.** Use of a κOR-ICL3 insertion with anti-ICL3 Nb6 enabled the 2.4 ​Å-resolution reconstruction of ∼46 ​kDa NTSR1 (EMD-26589, PDB:7UL2). κOR-ICL2 is a two-point insertion between two anti-parallel α-helices of the target protein, and is bound by a Nb (Nb6). Left of each panel: Schematics of assemblies. Middle: Colored electron density maps of full assemblies. Right: Zoom-ins of area indicated with black dashed line in middle panels. Colored dashed lines in panels c–d denote unresolved fusion connections. The molecular weight of the target protein (MW_target_) and the total assembly (MW_total_) are indicated above the schematics. Electron densities and structural models were produces using UCSF ChimeraX (Goddard et al., 2018). Note that the local target resolution may differ from the published global resolutions indicated here.

A nanobody (Nb) is smaller (∼12 ​kDa), internally more rigid than a Fab, and typically locks a target protein in a specific conformation (Zimmermann et al., 2018; Uchański et al., 2020; Goutam et al., 2022) thereby potentially reducing target flexibility. This locking feature can also be utilized to capture specific conformational states or weak protein-protein interactions. Because of their limited addition of MW, however, Nbs are often deployed in complex with other proteins. For instance, Bloch and co-workers directed a Fab fragment against a conserved surface in Nbs, dubbing it ‘NabFab’ (Bloch et al., 2021). Subsequently, they increased the rigidity of the Fab by directing a Fab-targeting Nb. This complex formed a stable fiducial marker and aided in structure determination of two 50-kDa transmembrane proteins, a bacterial MATE transporter (VcNorM) at 3.5 ​Å and a bacterial divalent metal ion transporter (ScaDMT) at 3.8 ​Å resolution (Fig. 2a).

Similarly, a conserved β-turn within a nanobody was fused to the adhesin domain of *Helicobacter pylori* (HopQ) and a *Escherichia coli* K12 glucosidase (YgjK), to produce 50–100 ​kDa ‘Megabodies’ (Mb) (Uchański et al., 2021). Mbs are modular as the fused Nb can be exchanged. Moreover, Mbs can be directly matured with yeast and phage display technology. Recently, a Mb was used as fiducial to allow structure determination of the hedgehog acetyltransferase transmembrane enzyme (HHAT, 62 ​kDa) to 2.7 ​Å resolution (Fig. 2b) (Coupland et al., 2021), and of the Na^+^–taurocholate co-transporting polypeptide (NTCP, 38 ​kDa) to 3.7 and 3.3 ​Å resolution using two different Mbs (Goutam et al., 2022). Aside from this success with fiducial-assisted cryo-EM on a small protein, Mbs have also been effective in solving preferred orientation issues. In addition, Mbs were developed that target nanodisc-forming membrane scaffold proteins (MSP) for general membrane protein-targeting (Uchański et al., 2021).

Even more complex assemblies were realized in which a Nb is stably bound by an anti-Nb Fab on one side, and by domain C of protein A of *Staphylococcus aureus* (PrAC) fused with a maltose binding protein (MBP) on the other side. This PrAC-MBP is extended with the Fab-binding protein AD (PrAD) and protein G (PrG) domains to stabilize the assembly, together referred to as ‘Legobodies’. Legobodies facilitated structure determination of the 22-kDa SARS-CoV-2 receptor binding domain (RBD) (Fig. 2c) at 3.6 ​Å and the small membrane KDEL receptor (23 ​kDa) at 3.2 ​Å (Wu and Rapoport, 2021). Like NabFabs and Mbs, Legobodies are relatively straight-forward to implement once a rigid target-specific Nb is available.

## 3 Target fusion strategies

To increase the MW of small proteins, several fusion strategies have also been developed. These approaches utilize, for instance, small epitope motifs to which then high-affinity Fabs are available, or large multimerizing proteins. For obtaining high resolution, the fusion with the target protein should be very rigid. A common tactic to achieve this rigidity is to extend C- or N-terminal α-helices. For cryo-EM SPA, however, a single connection through an extended α-helix is often still too flexible for high-resolution reconstruction (Mukherjee et al., 2020; Zhang, 2022b). Therefore, additional interactions between the fusion segment and target protein are typically required. We now discuss examples in which the target protein is covalently fused by two or three points of attachment.

Duan and co-authors have realized a fusion strategy that harnesses innate protein-protein interactions to render the size and morphology of the resulting complex compatible with SPA (Duan et al., 2020). They stabilized a target-ligand complex by fusing a so-called LgBit fragment of a split luciferase to the target protein and a LgBit-complementary HiBit peptide fragment to the ligand, creating a ‘NanoBit’. This two-point strategy has been particularly useful in - but probably not limited to – resolving G protein-coupled receptors (GPCRs) in complex with their G protein, such as the 3.0 ​Å reconstruction of the 46-kDa glucagon-like peptide 2 receptor (GLP-2R) (Fig. 2d) (Duan et al., 2020; Sun et al., 2020; Xia et al., 2021)

Another broadly applicable approach is to fuse epitopes to the target protein, thereby enabling subsequent binding of a Fab or Nb for fiducial-assisted SPA. Epitope tags as short as 14 amino acids have been tried on <50 ​kDa proteins with varying success (McIlwain et al., 2021; Tamura-Sakaguchi et al., 2021). An engineered thermostable variant of cytochrome *b*562 RIL (BRIL) has been most effective so far through its two-point insertion between two α-helices of target proteins. The BRIL element is recognized by an anti-BRIL Fab which can be used for fiducial-assisted SPA (Mukherjee et al., 2020). This strategy – with BRIL extending two α-helices by substituting the intracellular loop that initially connects them – facilitated structure determination of a 65 ​kDa Frizzled5 receptor (Fzd5) (Tsutsumi et al., 2020) (Fig. 2e), the GPCR adenosine A_2A_ receptor (45 ​kDa) at <4 ​Å resolution (Zhang, 2022b), and an inactive Epstein-Barr virus-induced GPCR 2 (EBI2) at 3.0 ​Å (Chen et al., 2022). BRIL fusions, through an extension of single C- or N-terminal α-helices, were shown too flexible for fiducial-assisted SPA (Mukherjee et al., 2020; Zhang, 2022b), but were effective in overcoming aggregation problems in one instance (Deng et al., 2021).

Similar to the two-point BRIL insertion strategy, the 87-kDa smoothened receptor (SMO) GPCR was resolved at 3.7 ​Å resolution by fusing a thermostable glycogen synthase domain from Pyrococcus abyssi (PGS) via a two-point α-helical extension (Zhang, 2022b) (Fig. 2f). Although the extended α-helices were found to be nicked, the required rigidity of the PGS domain was rescued due to an additional hydrophobic interaction between PGS and SMO.

Also, the 39-kDa AmpC beta-lactamase was utilized to replace an intracellular loop of the inactive β1-adrenoreceptor (45 ​kDa), and facilitated structure determination at 3.6 ​Å (Collu et al., 2021). Notably, AmpC was selected as a rigid insertion candidate by searching the Protein Data Bank for structures with anti-parallel α-helices 11 ​Å apart, compatible with the distance between GPCR helices.

A 4.2 ​Å-resolution structure of an inactive β2-adrenoreceptor GPCR was acquired through a three-point fusion with calcineurin, specifically by an insertion between transmembrane helices TM5-TM6, and by extension of TM7 (Xu et al., 2022). A comparable three-point attachment was realized for a 67-kDa complex of human sodium-glucose transporter 2 (hSGLT2) with membrane-associated protein 17 (MAP17), and led to a reported 2.95 ​Å resolution reconstruction (Niu et al., 2022). Here, a hSGLT2-inserted GFP formed a stable interaction with the anti-GFP Nb that was added as a MAP17 extension. This GFP-Nb addition protruded from the nanodisc and added enough MW and features to ensure particle alignment. This approach might be applicable to monomeric proteins as well, similar to the three-point calcineurin attachment (Xu et al., 2022).

Most recently, Robertson and colleagues developed a universal fiducial for inactive state GPCRs by substituting the conserved intracellular loop 3 (ICL3) for the κ-opioid receptor (κOR) ICL3 that is recognized by a specific Nb (Nb6), as well as by a Nb6-derived Mb (Mb6) (Robertson et al., 2021). This resulted in the high-resolution reconstruction of three sub-70-kDa inactive GPCRs - namely neurotensin 1 (NTSR1) (Fig. 2g), μ-opioid receptor, and the Somatostatin receptor 2.

Fusion strategies have shown to be effective in determining the structure of small proteins if one or multiple α-helices can be rigidly extended, for instance using inserted epitopes such as BRIL or κOR ICL3. These approaches have mentionable potential for more general applications, as similar helical motifs are abundant among many other membrane proteins. However, it should be noted that even with increasingly accurate methods for protein structure prediction such as AlphaFold (Jumper et al., 2021) and RoseTTAFold (Baek et al., 2021), and improved molecular dynamics simulations to probe structural flexibility (Robertson et al., 2021), laborious experimental screening over multiple variants is typically required for obtaining a rigid connection (Mukherjee et al., 2020; Tsutsumi et al., 2020; Chen et al., 2022) while preserving protein function (Robertson et al., 2021). Target binding approaches also require extensive but often standardized screening to identify specific binders (Helma et al., 2015; Uchański et al., 2021). Compared to target fusion strategies, target binding techniques may lock proteins into certain conformational states but are less prone to interfere with native protein structure.

## 4 Symmetric scaffolding strategies

**Fig. 3:**
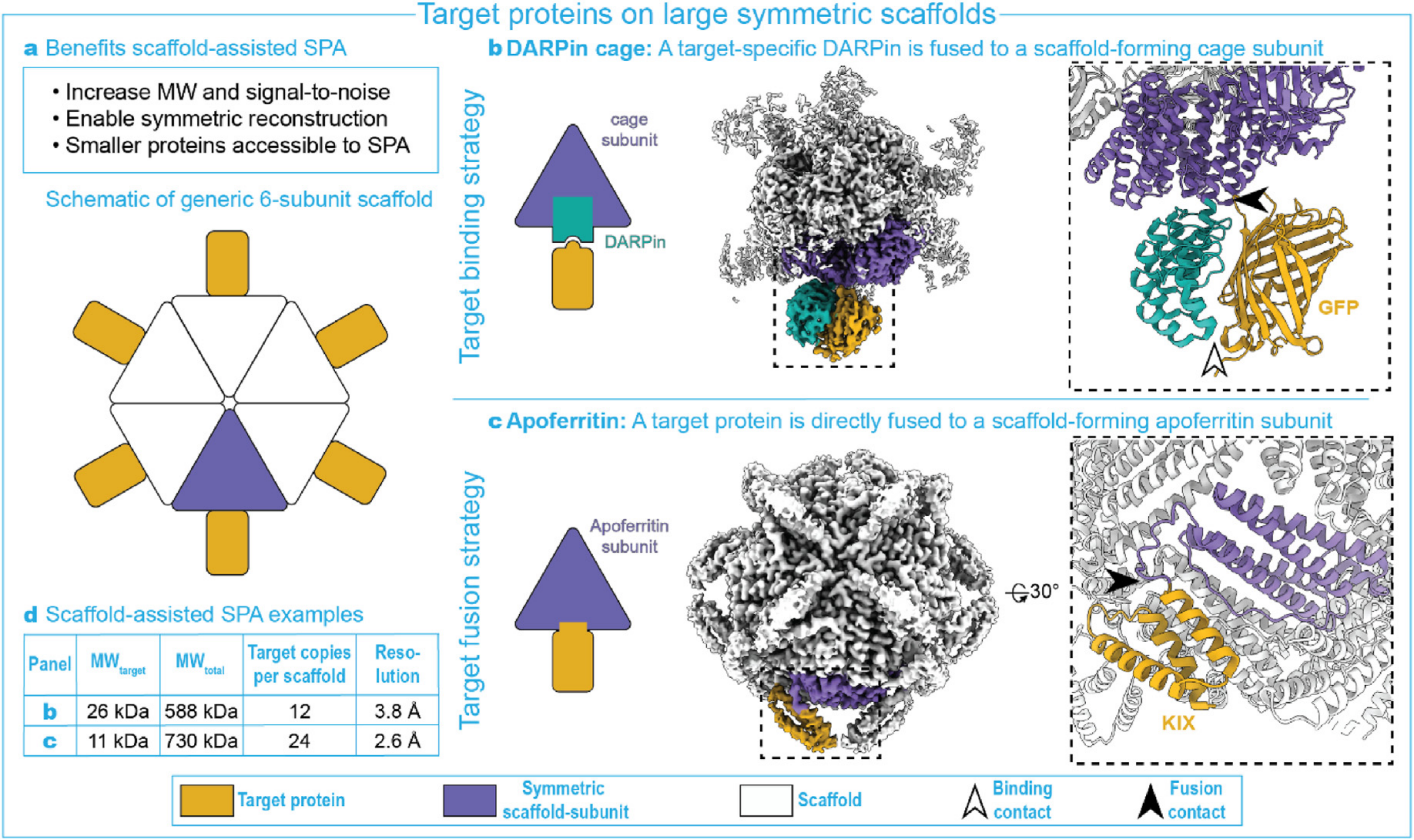
**a.** Benefits of using a symmetric scaffold for SPA as compared to non-scaffolding size augmentations. A schematic is shown of a generic symmetric scaffold with a single in-plane 6-fold symmetry axis and with target proteins docked at the periphery. **b.** Use of a target-specific DARPin fused with a cage subunits enabled the 3.8 ​Å-resolution reconstruction of 26-kDa GFP upon self-assembly of the DARPin-cage symmetric scaffold (EMD-9373 and EMD-9374, PDB:6NHV and 6NHT). **c.** Direct fusion with apoferritin enabled the 2.6 ​Å-resolution reconstruction of 11-kDa KIX domain upon self-assembly of the apoferritin symmetric scaffold (EMD-25791, PDB:7TB3. Left of panels b–c: Schematics of assemblies. Middle: Colored electron density maps of full assemblies. Right: Zoom-ins of area indicated with black dashed line in middle panels. **d.** Scaffold parameters corresponding to the examples given in panels b–c, including the molecular weight of the target protein (MW_target_) and the total assembly (MW_total_). Electron densities and structural models were produces using UCSF ChimeraX (Goddard et al., 2018). Note that the local target resolution may differ from the published global resolutions indicated here.

So far, we have discussed instances where small protein target binding and target fusion led to a direct increase in MW of the targets. Here we discuss examples in which the appended units organize into symmetric scaffolds achieving at least 20-fold MW-increase, with regularly spaced target proteins docked at the scaffold's periphery (Fig. 3a).

Liu and co-authors fused an anti-GFP DARPin to one of the subunits of a symmetric protein cage (Liu et al., 2018; Liu et al., 2019). DARPins are small (14 ​kDa) single-domain adapter proteins that consist of short α-helices separated by ankyrin repeat loops. The resulting ∼588 ​kDa symmetric cage-like assembly was able to bind 12 copies of GFP (26 ​kDa) simultaneously (Fig. 3b,d). This scaffold was easily distinguished from the background and its 12-copy binding additionally overcame the cryo-EM SPA specific problem of preferred orientation, allowing structure determination at 3.8 ​Å resolution for GFP. Designing DARPin-based scaffolds is not trivial, however. Flexibilities in the DARPin-target binding prevented Yao and colleagues from reaching sub-4 Å resolution for GFP with another scaffold design, even though their scaffolding complex itself could be resolved at 3 ​Å (Yao et al., 2019). Subsequently, the DARPin motif was dimerized to increase stability of the complex (Vulovic et al., 2021). In this case, issues with preferred orientation limited the resolution to 4.8 ​Å for GFP. Dimerization did improve stability of the complex, since a 4 ​Å-resolution structure for a 66 ​kDa human serum albumin (HSA) could be obtained.

A symmetric scaffolding approach relying on a single fusion was used to resolve the 11-kDa KIX domain of the CREB-binding protein (Fig. 3c-d) (Zhang, 2022a). Zhang and colleagues fused the KIX domain, through a single-point α-helical extension, rigidly to apoferritin – a protein that self-assembles into a 24-subunit symmetric shell, allowing KIX structure determination at 3–4 ​Å resolution. It should be noted that two cysteine mutations and a deletion of a flexible segment of the KIX motif were needed to achieve the required stability for high-resolution structure determination.

Due to the additional benefits of increased size and symmetric features, on top of their modular design, symmetric scaffolds are currently being explored as a universal method for small-protein cryo-EM SPA (Liu et al., 2019; Yeates et al., 2020).

## 5 Summary

**Fig. 4:**
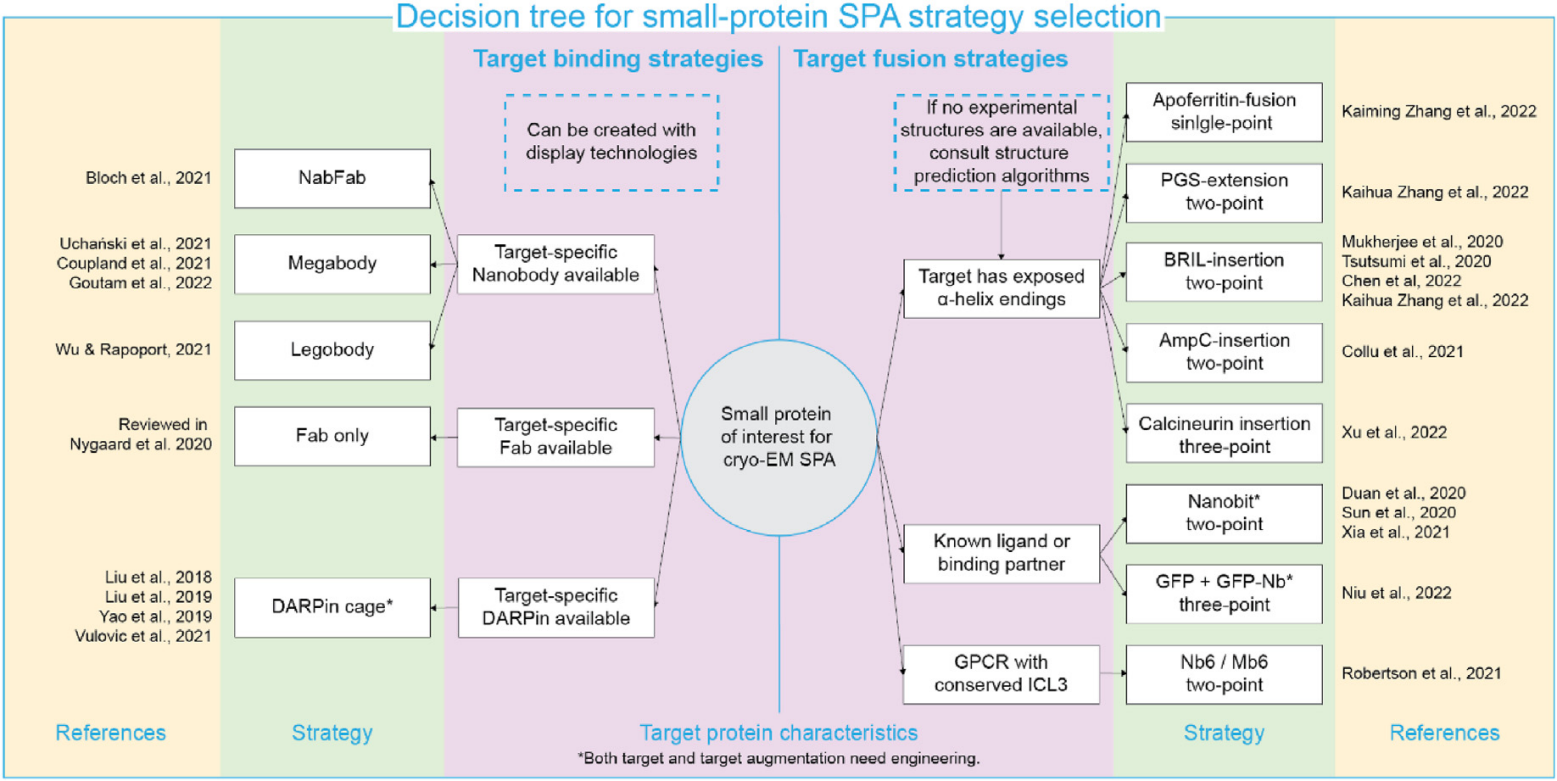
To enable cryo-EM SPA of small target proteins of interest (center vignette), the characteristics of the protein can guide choice of strategy for protein size-augmentation. A target binding (left) or a target fusion (right) strategy can be employed, each having multiple variants that together cover a wide range of small protein targets. Boxes in dashed blue lines are additional suggestions. (For interpretation of the references to colour in this figure legend, the reader is referred to the Web version of this article.)

In summary, various techniques have become available in recent years to extend structure determination by cryo-EM SPA into the realm of small proteins (<100 ​kDa), which make up the vast majority of the human proteome. Size-dependent resolution limits can be overcome by clever size augmentation approaches, such as Nb-based assemblies, fused Fab epitopes, and symmetric scaffolding. The effectiveness of all above-mentioned approaches mainly depends on the rigidity of the resulting complex (Mukherjee et al., 2020; Tsutsumi et al., 2020; Zhang, 2022b), for which predictive computational approaches are recently emerging (Baek et al., 2021; Jumper et al., 2021; Robertson et al., 2021). In Fig. 4, we offer an overview of the discussed approaches, including considerations and suggestions for employing target protein binding versus target protein fusion strategies.

## Authorship contributions

**Category 1**.

Conception and design of study: KW, CG; acquisition of data: analysis and/or interpretation of data: KW, CG, DHM,

**Category 2**.

Drafting the manuscript: KW, CG; revising the manuscript critically for important intellectual content: CG, DHM.

**Category 3**.

Approval of the version of the manuscript to be published: KW, CG, DHM.

## Declaration of competing interest

The authors declare that they have no known competing financial interests or personal relationships that could have appeared to influence the work reported in this paper.

## Data Availability

No data was used for the research described in the article.
